# Inhibition of cGMP-metabolizing PDEs as target for cognitive enhancement

**DOI:** 10.1186/2050-6511-16-S1-A19

**Published:** 2015-09-02

**Authors:** Holger Rosenbrock

**Affiliations:** 1 Department of CNS Diseases Research, Boehringer Ingelheim Pharma GmbH & Co KG, Bibearch, Germany

## Background

Inhibition of specific phosphodiesterases (PDEs) in the brain has gained attention as a potential new approach for memory enhancement. Among those PDEs are PDE2A and PDE9A which are either, in case of PDE2A, dual-specific for cyclic guanosine and cyclic adenosine monophosphate (cGMP and cAMP, respectively) or selective for cGMP, in case of PDE9A. Both PDEs are expressed in cognition relevant regions of the brain, and in these regions, especially cGMP plays an important role as second messenger in neurons related to NMDA receptor signalling facilitating synaptic plasticity and memory formation. Thus, PDE2A and PDE9A inhibitors are hypothesized to improve cognitive function via improving glutamatergic neurotransmission and/or signalling pathways leading to strengthening of synaptic plasticity. In order to characterize mechanistically the effects of PDE2A and PDE9A, selective inhibitors of both PDEs were tested on long-term potentiation (LTP) in rat hippocampal slices, a widely accepted cellular experimental model of synaptic plasticity and memory formation as well as on hippocampal paired-pulse facilitation (PPF), a model to assess presynaptic function. Furthermore, both inhibitors were evaluated regarding enhancement of cyclic nucleotide levels in brain and memory performance in rodent cognition tasks.

## Results

Both, the PDE2A inhibitor PF-999 [1] and the PDE9A inhibitor Bay 73-6691 [2] increased hippocampal LTP, but in contrast to PDE2A inhibition, PDE9A inhibition not only enhances early and late LTP, but even transforms early LTP into late LTP. Regarding effects on hippocampal PPF, only the PDE2A inhibitor but not the PDE9A inhibitor was able to modulate presynaptic function, indicating that PDE2A is localized at the presynaptic side whereas PDE9A might be located at the postsynaptic side.

Both inhibitors showed a dose-dependent increase of cGMP levels in mouse hippocampus after oral application, but exhibited no effect on cAMP levels. Memory performance could be improved by both inhibitors in the T-maze continuous alternation task using pharmacologically impaired mice or in the object location task using APP-transgenic Tg2576 mice.

## Conclusion

PDE2A and PDE9A inhibition led to an increase of LTP in rat hippocampal slices. Corroborating previous reports on functional effects of other PDE2A and PDE9A inhibitors, these data demonstrate that inhibition of these PDEs may be an effective way of increasing glutamatergic neurotransmission/ signalling and of strengthening synaptic plasticity. Systemic administration of both compounds led to an increase in cGMP levels in the mouse hippocampus demonstrating functional target engagement, i.e. PDE2A or PDE9A inhibition in the brain. In accordance with recent data on memory enhancing efficacy of other PDE2A or PDE9A inhibitors in rodents, our data further demonstrates that PDE2A and PDE9A inhibition may be a potential approach to pharmacologically improve cognition in CNS disorders.
